# Sex differences in associations between creatinine and cystatin C-based kidney function measures with stroke and major bleeding

**DOI:** 10.1177/23969873231173282

**Published:** 2023-05-12

**Authors:** Jennifer Susan Lees, Nicole L De La Mata, Michael K Sullivan, Melanie L Wyld, Brenda M Rosales, Rachel Cutting, James Alan Hedley, Elaine Rutherford, Patrick Barry Mark, Angela C Webster

**Affiliations:** 1 School of Cardiovascular and Metabolic Health, University of Glasgow, Glasgow, UK; 2 School of Public Health, Faculty of Medicine and Health, University of Sydney, Camperdown, NSW, Australia; 3 Renal Unit, Mountainhall Treatment Centre, NHS Dumfries and Galloway, Dumfries, UK

**Keywords:** Chronic kidney disease, CKD, stroke, bleeding, cystatin C, risk

## Abstract

**Purpose:**

We sought to explore whether adding kidney function biomarkers based on creatinine (eGFR_Cr_), cystatin C (eGFR_Cys_) or a combination of the two (eGFR_Cr-Cys_) could improve risk stratification for stroke and major bleeding, and whether there were sex differences in any additive value of kidney function biomarkers.

**Method:**

We included participants from the UK Biobank who had not had a previous ischaemic or haemorrhagic stroke or major bleeding episode, and who had kidney function measures available at baseline. Cause-specific Cox proportional hazards models tested associations between eGFR_Cr_, eGFR_Cys_ and eGFR_Cr-Cys_ (mL/min/1.73 m^2^) with ischaemic and haemorrhagic stroke, major bleeding (gastrointestinal or intracranial, including haemorrhagic stroke) and all-cause mortality.

**Findings:**

Among 452,879 eligible participants, 246,244 (54.4%) were women. Over 11.5 (IQR 10.8–12.2) years, there were 3706 ischaemic strokes, 795 haemorrhagic strokes, 26,025 major bleeding events and 28,851 deaths. eGFR_Cys_ was more strongly associated with ischaemic stroke than eGFR_Cr_: an effect that was more pronounced in women (men – HR: 1.16, 95% CI: 1.12–1.19; female to male comparison – HR: 1.11, 95% CI: 1.05–1.16, per 10 mL/min/1.73 m^2^ decline in eGFR_Cys_). This interaction effect was also demonstrated for eGFR_Cr-Cys_, but not eGFR_Cr_. eGFR_Cys_ and eGFR_Cr-Cys_ were more strongly associated with major bleeding and all-cause mortality than eGFR_Cr_ in both men and women. Event numbers were small for haemorrhagic stroke.

**Discussion:**

To a greater degree than is seen in men, eGFR_Cr_ underestimates risk of ischaemic stroke and major bleeding in women compared to eGFR_Cys_. The difference between measures is likely explained by non-GFR biology of creatinine and cystatin C.

**Conclusion:**

Enhanced measurement of cystatin C may improve risk stratification for ischaemic stroke and major bleeding and clinical treatment decisions in a general population setting, particularly for women.

## Graphical abstract

**Graphical abstract:**
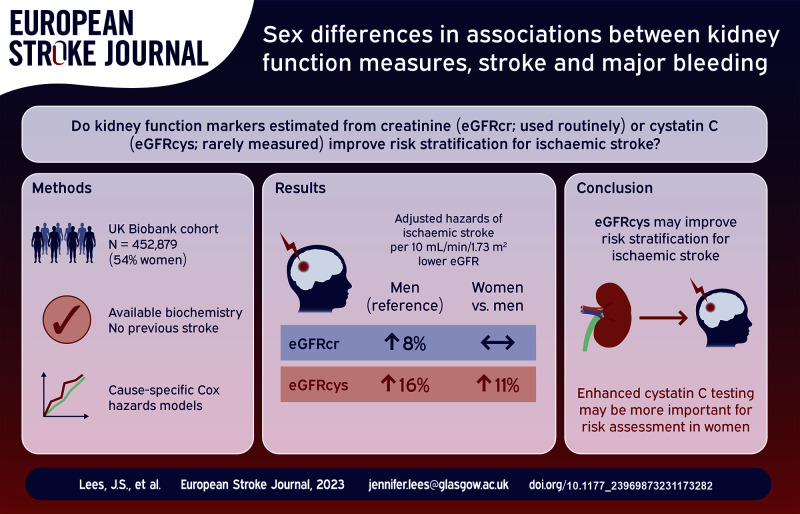

## Background

Stroke is a leading cause of severe adult disability and death globally.^1^ Stroke risk increases linearly with worsening kidney function. The increase in magnitude of stroke risk is evident even in people with mild changes in kidney function (estimated glomerular filtration rate, eGFR: 60–90 mL/min/1.73 m^2^),^2,3^ and becomes more pronounced in people with advanced chronic kidney disease (CKD), including kidney failure. CKD is associated with development of stroke-specific risk factors such as heart failure^4^ and atrial fibrillation,^4^ and an increased likelihood of bleeding due to platelet dysfunction, anaemia, altered drug pharmacokinetics and pharmacodynamics.^5^ Despite this, kidney function is not routinely considered in many risk-assessment tools used to guide primary prevention of cardiovascular events including ischaemic stroke.^6–8^ Among people with CKD, risk prediction tools typically underestimate the risk of both ischaemic stroke^9^ and major bleeding.^10^

Kidney function is estimated from serum biomarkers. Serum creatinine is the most widely used biomarker to estimate kidney function (eGFR_Cr_). However, cystatin C is an alternative biomarker of kidney function (eGFR_Cys_) and can be used in combination with creatinine (eGFR_Cr-Cys_). Incorporating cystatin C into kidney function estimation is thought to improve accuracy compared with creatinine alone. Compared with eGFR_Cr_, eGFR_Cys_ and eGFR_Cr-Cys_ better identify individuals at the highest level of risk of composite cardiovascular disease^11,12^ and could be used to guide treatment selection.^11,13,14^

We sought to explore: (i) whether adding kidney function biomarkers would improve risk prediction for ischaemic stroke and major bleeding; and – knowing that stroke risk and kidney function differ by sex – (ii) whether kidney function biomarkers improved risk prediction for stroke and major bleeding to a greater extent in men or women in the UK Biobank.

## Methods

### Participants and population

We included all consenting participants in the UK Biobank who had not had a previous ischaemic stroke, haemorrhagic stroke or other major bleeding episode, and who had available biochemistry measures at baseline to estimate kidney function. Of 502,536 participants aged 37–73 initially recruited, we excluded 76 who withdrew ongoing consent for follow-up; 33,491 participants with missing biochemistry data at baseline; 544 with kidney failure at baseline; and 15,546 with a previous history of ischaemic or haemorrhagic stroke or major bleeding, leaving 452,879 participants in our cohort.

Details of the recruitment procedure and protocol to the UK Biobank cohort have been described previously.^15,16^ In brief, the UK Biobank invited participation by post from adults (target age 40–69 years) registered with the National Health Service (NHS) between 2006 and 2010, and who lived within reasonable travelling distance of an assessment centre in the United Kingdom. Participants provided written informed consent for baseline phenotyping and prospective follow-up, with electronic linkage to hospital health records and death registries, allowing extraction of diagnoses from these records using International Classification of Disease and Related Health Problems (ICD) 10th Revision 2016 (ICD-10) codes. Ethical approval was granted from the North West Multi-Centre Research Ethics Committee (REC reference 11/MW/03820). The study was conducted under UK Biobank project code 69,891 and reported according to STROBE principles.

### Clinical variables

We used a combination of self-report, and administrative health records to classify presence or absence of clinical variables. Medical history of cardiovascular disease and diabetes was recorded in a self-reported health questionnaire. Presence of atrial fibrillation/flutter (AF) and heart failure at baseline was defined as self-reported previous history of AF, or presence of ICD-10 code ‘I48’ from any hospital episode prior to the assessment date for UK Biobank. Presence of heart failure at baseline was defined as the presence of ICD-10 code ‘I50’ from any hospital episode prior to the assessment date for UK Biobank. There were no additional cases of AF or heart failure identified from hospital episode statistics ICD 9th Revision (ICD-9) codes used prior to 2016.

Sex of the participant (as assigned at birth) was acquired from central NHS registry at recruitment, but was updated by the participant in a minority of cases. Ethnicity was self-selected from a hierarchical tree structure based on UK census data, and included the following initial categories: White, Mixed, Asian or Asian British, Black or Black British, Chinese, Other ethnic group, Do not know, Prefer not to answer. Smoking history was self-reported from the following options: Never, Previous, Current or Prefer not to answer.

A single baseline estimate of kidney function was calculated from serum creatinine (eGFR_Cr_), cystatin C (eGFR_Cys_) or the combination of creatinine and cystatin C (eGFR_Cr-Cys_) using the CKD-EPI equations 2009^17^ (as these are the equations currently recommended for use in clinical practice in the UK).^18^ Each eGFR is measured in mL/min/1.73 m^2^ and on a scale from 0 mL/min/1.73 m^2^ (indicating no kidney function, or kidney failure) to over 90 mL/min/1.73 m^2^ (indicating normal kidney function, in the absence of albuminuria). Moderate to advanced CKD is considered to exist when eGFR is <60 mL/min/1.73 m^2^; however, changes in kidney function above or below these ranges can still be clinically significant and associated with future risk of disease. On average, eGFR_Cys_ and eGFR_Cr-Cys_ tend to provide lower estimates of kidney function than eGFR_Cr_, and differences can vary by sex (Supplemental Figure S1).

### Outcomes of interest

1. Ischaemic stroke (fatal or non-fatal) was pre-defined by UK Biobank. In brief, this algorithm incorporates self-reported medical history (collected at baseline), relevant ICD-9 and ICD-10 codes identified on any diagnosis from hospital episode statistics or death certification. The date and source of ischaemic stroke were provided in data fields supplied by the UK Biobank. Full details of the methodology to identify ischaemic stroke can be found in the published UK Biobank algorithm.^19^
2. Haemorrhagic stroke (fatal or non-fatal) was pre-defined by UK Biobank and collected from linked hospital episode statistics and/or death certification as for ischaemic stroke. Full details of the methodology to identify ischaemic stroke can be found in the published UK Biobank algorithm.^19^
3. Major bleeding (gastrointestinal or intracranial bleeding, including haemorrhagic stroke) was defined from hospital episode statistics and death certification using the following ICD-10 codes: I60, I61, I62, I85.0, I98.3, K22.6, K25.0, K25.2, K25.4, K25.6, K26.0, K26.2, K26.6, K27.0, K27.2, K27.4, K27.6, K28.0, K28.2, K28.4, K28.6, K29.0, K62.5, K66.1, K76.2, K92.0, K92.1, K92.2, S06.4, S06.5, S06.6.^20^ This composite bleeding outcome was included in this form to test discrimination of risk prediction tools for major bleeding in anticoagulated participants with AF (see ‘Analysis’ section below).
4. All-cause mortality was defined from the date of death from linked death certification records according to a pre-specified algorithm.^19^

Participants were followed from enrolment in the UK Biobank until the first occurrence of an outcome of interest, death or the end of data collection (31st August 2020).

### Analysis

Baseline patient characteristics and clinical data were summarised as mean (standard deviation: SD), median (interquartile range: IQR) or count (percentage: %) and compared using *t*-test or Wilcoxon rank sum test for continuous variables or, chi-square test for categorical variables, as appropriate.

Missing data in UK Biobank were minimal and assumed to be missing at random. Where data were missing, we undertook multiple imputation by chained equations over five iterations.

Cumulative incidence of ischaemic stroke, haemorrhagic stroke, major bleeding and all-cause mortality was assessed and compared in women versus men.

Association of kidney function measures with outcomes (fatal/non-fatal ischaemic stroke, haemorrhagic stroke, major bleeding and all-cause mortality), were tested in nested, cause-specific Cox proportional hazards models, censored for death from other causes. The Cox proportional hazards assumption was checked by visual inspecting Schoenfeld residuals. Model 1 was adjusted for accepted risk factors for ischaemic stroke: age, smoking, systolic and diastolic blood pressure, medications for blood pressure or cholesterol, baseline total, LDL and HDL cholesterol, pre-existing heart failure, pre-existing AF or other atherosclerotic cardiovascular disease (including myocardial infarction and peripheral vascular disease). For haemorrhagic stroke and major bleeding, Model 1 was adjusted for accepted risk factors for major bleeding: age, history of or medication for hypertension, systolic or diastolic blood pressure, haemoglobin and haematocrit. For both outcomes, Model 2 was adjusted for variables included in Model 1, plus prescription of an anticoagulant (warfarin or low-molecular weight heparin) or an antiplatelet agent (aspirin, clopidogrel, prasugrel or dipyridamole) at baseline. Of note, due to the recruitment period of UK Biobank (2006–2010), no patient was prescribed other antiplatelets or anticoagulants now used routinely in clinical practice (including ticagrelor and direct oral anticoagulant medications). Model 3 was adjusted for variables included in Model 2, plus ethnicity, body mass index and hip to waist ratio, as these are variables that may affect the reliability of creatinine and/or cystatin C as markers of kidney function. In all models, we initially tested for an interaction between kidney function measures and sex. Subsequent analyses were then stratified by sex.

To test the performance of validated risk prediction models for ischaemic stroke and major bleeding in AF, additional analyses were conducted in a subgroup of participants with AF at baseline. First, we repeated cause-specific Cox proportional hazards models within this subgroup, as for the whole population. Second, we assessed whether inclusion of eGFR_Cr_, eGFR_Cys_ or eGFR_Cr-Cys_ could improve accuracy of risk discrimination of ischaemic and major bleeding – compared to established risk prediction tools – using area under receiver operating curves (AUROC). We used risk prediction tools that are currently recommended by the National Institute for Health and Care Excellence (NICE) guidelines for diagnosis and management of atrial fibrillation (NG196).^21^ For ischaemic stroke, this was the ‘CHADS-VASC’ score^7^: a tool that scores risk of stroke in the following year according to the presence (or absence) of known risk factors for ischaemic stroke, including heart failure, hypertension, age, diabetes, vascular disease, sex and history of stroke (range 0–9; low to high). For major bleeding, the reference standard was the ‘ORBIT’ score, developed from the national Outcomes Registry for Better Informed Treatment of Atrial Fibrillation.^22^ ORBIT provides a points-based score based on age, haemoglobin, haematocrit, bleeding history, insufficient kidney function (eGFR < 60 mL/min/1.73 m^2^) and treatment with an antiplatelet agent (range 0–7; low to high).

To assess the added value of kidney function markers for risk prediction of ischaemic stroke, the base model included variables in the CHADS-VASC score, excluding sex (as analyses were stratified by sex) and history of stroke (as participants with previous stroke were excluded). The base model for major bleeding including variables in the ORBIT score, but did not include eGFR < 60, to allow comparisons between eGFR_Cr_ with eGFR_Cys_ and eGFR_Cr-Cys_ on discriminative performance for major bleeding. We tested discrimination of the CHADS-VASC and ORBIT base models with addition of eGFR_Cr_, eGFR_Cys_ or eGFR_Cr-Cys_.

Analyses were conducted R statistical software version 4.2.1 in RStudio for Mac version 2021.09.0 using *tidyverse, mice, pROC, survival, ggplot2* and *ggpubr* packages.

## Results

### Whole population

**Figure 1.**
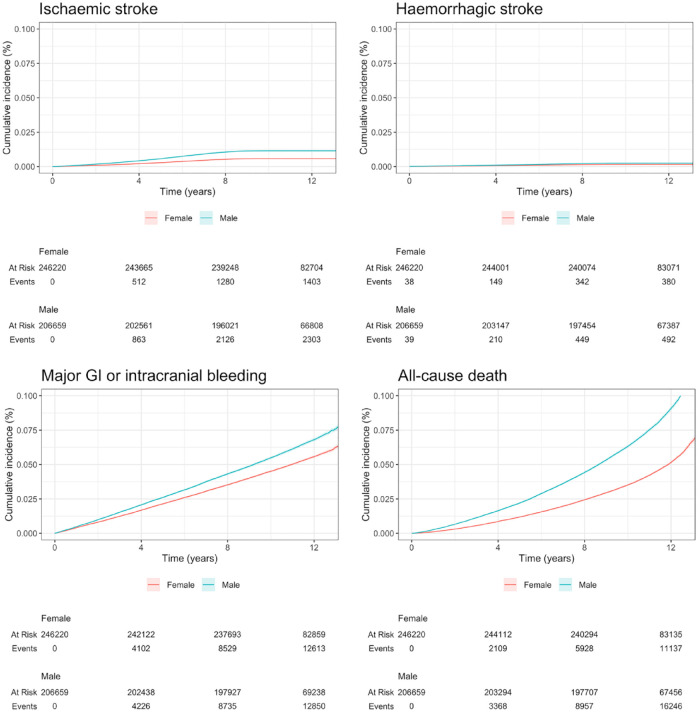
Cumulative incidence (%) of fatal/non-fatal ischaemic stroke censored for non-stroke death (top left), haemorrhagic stroke censored for non-stroke death (top right), major gastrointestinal (GI) or intracranial bleeding censored for non-bleeding-related death (bottom left) and all-cause mortality (bottom right), stratified by sex.

Among 452,879 eligible participants, 206,698 (45.6%) were men with median age 58 (IQR 50–64) years; 246,244 (54.4%) were women with median age 57 (IQR 60–63) years (Table 1). Baseline kidney function was similar between women and men (eGFR_Cr_ 93 (IQR 83–95) vs 93 (IQR 83–94) mL/min/1.73 m^2^, respectively). Over median follow-up of 11.5 (IQR 10.8–12.2) years (similar in men and women), there were 3706 fatal/non-fatal ischaemic strokes (1403 in women; 2303 in men), 795 haemorrhagic strokes (342 in women; 453 in men), 26,025 fatal/non-fatal major bleeding events (12,886 in women; 13,139 in men) and 28,851 deaths from any cause. Cumulative incidence of ischaemic stroke, haemorrhagic stroke, major bleeding and all-cause mortality were lower in women than in men (Figure 1).

**Table 1.** Baseline characteristics in full population.

	Female	Male	*p*
*N*	246,220	206,659	
Age (median [IQR])	57.00 [50.00, 63.00]	58.00 [50.00, 64.00]	<0.001
Ethnicity: *n* (%)			<0.001
White	232346 (94.4)	194620 (94.2)	
Mixed	617 (0.3)	357 (0.2)	
Black	4622 (1.9)	3316 (1.6)	
South Asian	4471 (1.8)	5013 (2.4)	
Chinese	887 (0.4)	520 (0.3)	
Other	2303 (0.9)	1714 (0.8)	
Unknown	974 (0.4)	1119 (0.5)	
Smoking: *n* (%)			<0.001
Never	146570 (59.5)	101316 (49.0)	
Previous	76959 (31.3)	78999 (38.2)	
Current	21741 (8.8)	25493 (12.3)	
Unknown	950 (0.4)	851 (0.4)	
Systolic BP (mean (SD))	137.18 (20.22)	142.72 (18.58)	<0.001
Diastolic BP (mean (SD))	80.68 (10.57)	84.03 (10.56)	<0.001
Body mass index (mean (SD))	27.04 (5.16)	27.82 (4.23)	<0.001
Hip:waist ratio (mean (SD))	1.23 (0.10)	1.07 (0.08)	<0.001
cr_cat (%)			<0.001
eGFR_Cr_ > 90–105	115472 (46.9)	95467 (46.2)	
eGFR_Cr_ > 105	32494 (13.2)	25289 (12.2)	
eGFR_Cr_ > 75–90	67433 (27.4)	60675 (29.4)	
eGFR_Cr_ > 60–75	25522 (10.4)	20757 (10.0)	
eGFR_Cr_ > 45–60	4572 (1.9)	3660 (1.8)	
eGFR_Cr_ > 30–45	608 (0.2)	641 (0.3)	
eGFR_Cr_ ⩽30	119 (0.0)	170 (0.1)	
eGFR_Cr_ (mL/min/1.73 m^2^) (mean (SD))	93.22 [83.00, 100.40]	92.46 [83.03, 99.75]	<0.001
eGFR_Cys_ (median [IQR])	90.39 [77.86, 101.38]	88.34 [77.13, 100.25]	<0.001
eGFR_Cr-Cys_ (mL/min/1.73 m^2^) (mean (SD))	91.88 [81.67, 101.21]	90.69 [81.37, 99.69]	<0.001
Urine albumin:creatinine ratio (median [IQR])	0.00 [0.00, 0.55]	0.00 [0.00, 0.62]	<0.001
Total cholesterol (mean (SD))	5.88 (1.12)	5.49 (1.13)	<0.001
LDL cholesterol (mean (SD))	3.63 (0.87)	3.49 (0.86)	<0.001
HDL cholesterol (mean (SD))	1.60 (0.38)	1.28 (0.31)	<0.001
C-reactive protein (mg/L) (mean (SD))	2.69 (4.32)	2.45 (4.32)	<0.001
Haemoglobin (g/dL) (mean (SD))	13.50 (0.97)	14.99 (1.02)	<0.001
Haematocrit (%) (mean (SD))	39.23 (2.82)	43.29 (2.99)	<0.001
Diabetes: *n* (%)	8986 (3.6)	14058 (6.8)	<0.001
Hypertension: *n* (%)	54485 (22.1)	53304 (25.8)	<0.001
Cardiovascular disease: *n* (%)	1843 (0.7)	2167 (1.0)	<0.001
Heart failure: *n* (%)	570 (0.2)	1760 (0.9)	<0.001
Atrial fibrillation: *n* (%)	1943 (0.8)	4588 (2.2)	<0.001
Medications for blood pressure: *n* (%)	25106 (10.2)	20345 (9.8)	<0.001
Medications for cholesterol: *n* (%)	30302 (12.3)	46183 (22.3)	<0.001
Anticoagulant: *n* (%)	1292 (0.5)	2995 (1.4)	<0.001
Antiplatelet: *n* (%)	23988 (9.7)	38505 (18.6)	<0.001

Overall, women displayed fewer cardiometabolic risk factors: women were less likely to be current or previous smokers, had lower systolic and diastolic blood pressure, were less likely to have diabetes, hypertension, cardiovascular disease, AF and heart failure at baseline, and were less likely to be treated with statins (reflected in slightly higher total, LDL and HDL cholesterol), antiplatelets or anticoagulant medications (Table 1). The number of events by eGFR category are displayed for eGFR_Cr_ in Table 2, for eGFR_Cys_ in Supplemental Table S1 and for eGFR_Cr-Cys_ in Supplemental Table S2.

**Table 2.** Events of interest by estimated glomerular filtration rate based on serum creatinine (eGFR_Cr_) category.

Sex	eGFR_Cr_ category (mL/min/1.73 m^2^)	*N*	Ischaemic stroke (*N*=)	Haemorrhagic strokes (*N*=)	Major bleed (*N*=)	All-cause mortality (*N*=)
Male	>105	25,289	140	33	1457	1239
	>90–105	95,467	953	174	5720	7003
	>75–90	60,675	724	150	3882	5321
	>60–75	20,757	334	66	1561	2427
	>45–60	3660	115	23	387	777
	>30–45	641	31	5	102	242
	⩽30	170	6	2	30	74
Female	>105	32,494	78	22	1484	755
	>90–105	115,472	558	154	5967	5222
	>75–90	67,433	432	91	3491	3351
	>60–75	25,522	254	59	1551	1738
	>45–60	4572	65	15	322	498
	>30–45	608	10	1	57	144
	⩽30	119	6	0	14	47

In Cox proportional hazards models, lower baseline eGFR was associated with higher risk of ischaemic stroke, major bleeding and all-cause mortality in both men and women. In both women and men, eGFR_Cys_ and eGFR_Cr-Cys_ were more sensitive in detecting increased hazards of these outcomes with small reductions from the reference (eGFR > 90–105). Compared with eGFR_Cr_, eGFR_Cys_ and eGFR_Cr-Cys_ were more strongly associated with risk of ischaemic stroke, major bleeding and all-cause mortality. Estimates provided for eGFR < 30 (all measures) are likely to be unreliable owing to small numbers of participants and events in this group.

For every 10 mL/min/1.73 m^2^ decline in eGFR_Cys_ below 90 mL/min/1.73 m^2^, ischaemic stroke risk increased more substantially in women compared to men (HR in men: 1.16, 95% CI: 1.12–1.19; HR in women: 1.28, 95% CI: 1.23–1.33; women to men comparison: HR: 1.11, 95% CI: 1.05–1.16; Table 3). This statistical interaction was present also for eGFR_Cr-Cys_, but crossed the null for eGFR_Cr_ (Table 3).

**Table 3.** Interaction effects between sex and kidney function measures for major outcomes.

	Female HR (95% CI)	Male HR (95% CI)	Female to male comparison HR (95% CI)
Ischaemic stroke			
eGFR_Cr_	1.15 (1.10–1.20)	1.08 (1.04–1.13)	1.06 (1.00–1.12)
eGFR_Cys_	1.27 (1.23–1.33)	1.16 (1.12–1.19)	1.11 (1.05–1.16)
eGFR_Cr-Cys_	1.25 (1.20–1.31)	1.14 (1.10–1.18)	1.10 (1.04–1.16)
Haemorrhagic stroke			
eGFR_Cr_	1.08 (0.97–1.20)	1.09 (1.00–1.19)	0.99 (0.87–1.13)
eGFR_Cys_	1.12 (1.02–1.22)	1.12 (1.04–1.21)	1.00 (0.89–1.11)
eGFR_Cr-Cys_	1.13 (1.03–1.25)	1.13 (1.04–1.22)	1.01 (0.89–1.14)
Major bleeding			
eGFR_Cr_	1.01 (0.99–1.03)	1.04 (1.02–1.06)	0.97 (0.95–0.99)
eGFR_Cys_	1.08 (1.07–1.10)	1.09 (1.08–1.11)	0.99 (0.97–1.01)
eGFR_Cr-Cys_	1.06 (1.04–1.08)	1.08 (1.06–1.10)	0.98 (0.96–1.00)
All-cause mortality			
eGFR_Cr_	1.09 (1.08–1.11)	1.09 (1.07–1.11)	1.00 (0.98–1.02)
eGFR_Cys_	1.26 (1.24–1.28)	1.26 (1.24–1.28)	1.00 (0.98–1.02)
eGFR_Cr-Cys_	1.22 (1.20–1.23)	1.21 (1.20–1.23)	1.00 (0.98–1.02)

Cox proportional hazards models for risk of outcomes of interest according to a 10 mL/min/1.73 m^2^ decline in each eGFR from a within-group reference value of 90 mL/min/1.73 m^2^. Models included a sex:eGFR interaction term, and are fully adjusted for age, pre-existing stroke, other vascular disease, hypertension or use of antihypertensives, diabetes, heart failure and atrial fibrillation, smoking status, systolic and diastolic blood pressure, medications for cholesterol, baseline total, HDL and LDL cholesterol, use of anticoagulant, antiplatelet, body mass index, hip-to-waist ratio and ethnicity.

In sex-stratified analyses, compared to the female reference group (eGFR > 90–105), ischaemic stroke risk was raised in women with eGFR_Cys_ (aHR: 1.56, 95% CI: 1.33–1.83) or eGFR_Cr-Cys_ (aHR: 1.68, 95% CI 1.43–1.97) >60–75 mL/min/1.73 m^2^, and was doubled in women with eGFR_Cys_ > 45–60 mL/min/1.73 m^2^ (eGFR_Cys_ – aHR: 2.13, 95% CI: 1.73–2.62; eGFR_Cr-Cys_ – aHR: 1.92, 95% CI: 1.50–2.45).

**Figure 2.**
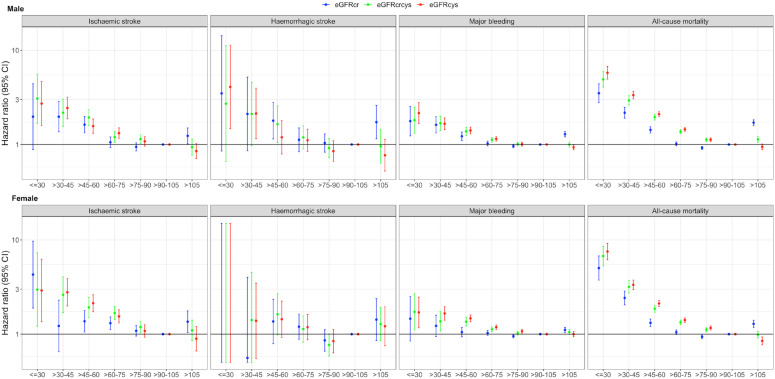
Forest plots displaying cause-specific hazard ratio and 95% confidence intervals (95% CI) for ischaemic stroke (left), haemorrhagic stroke (middle) or any major bleeding (right). Results taken from Cox proportional hazards models censored for death not caused by the outcome of interest. Ischaemic stroke models were adjusted for age, pre-existing stroke, other vascular disease, hypertension or use of antihypertensives, diabetes, heart failure and atrial fibrillation, smoking status, systolic and diastolic blood pressure, medications for cholesterol, baseline total, HDL and LDL cholesterol, use of anticoagulant, antiplatelet, body mass index, hip-to-waist ratio and ethnicity. Haemorrhagic stroke and major bleeding models were adjusted for age, hypertension or use of antihypertensives, systolic and diastolic blood pressure, baseline haemoglobin and haematocrit, use of anticoagulant, antiplatelet, body mass index, hip-to-waist ratio and ethnicity. All models were stratified by sex. The reference value was eGFR > 90–105 mL/min/1.73 m^2^ for all eGFR measures.

By comparison, eGFR_Cr_ detected increased risk in women, but at much smaller magnitude with eGFR > 45–60 (aHR: 1.37, 95% CI: 1.05–1.78), and did not detect an increase in risk among women with eGFR_Cr_ > 60–75 mL/min/1.73 m^2^. A similar pattern was observed in men, though with smaller discrepancy between aHR for the different eGFR measures (Figure 2 and Supplemental Tables S3 and S4).

There was at least a trend towards increased risk of haemorrhagic stroke in men and women with lower eGFR down to 45 mL/min/1.73 m^2^ (Figure 2 and Supplemental Tables S5 and S6). Estimates for eGFR below this level are difficult to interpret owing to very low numbers of events. There was no evidence of statistical interaction between sex and eGFR_Cr_, eGFR_Cys_ or eGFR_Cr-Cys_ for haemorrhagic stroke (Table 3).

With each 10 mL/min/1.73 m^2^ reduction in eGFR_Cr_ from 90 mL/min/1.73 m^2^, women were slightly less likely than men to suffer a major bleed (female to male comparison: HR: 0.97, 95% CI: 0.95–0.99). There was no statistical interaction between eGFR_Cys_ and sex, nor eGFR_Cr-Cys_ and sex, for major bleeding (Table 3).

In sex-stratified analyses and compared to the reference group (eGFR > 90–105), women with eGFR_Cys_ or eGFR_Cr-Cys_ < 75 were at mildly increased risk of major bleeding, and this pattern became more pronounced at lower eGFR_Cys_ or eGFR_Cr-Cys_. Women with eGFR_Cys_ or eGFR_Cr-Cys_ > 45–60 were at least 36% more likely to have a major bleed compared to the reference group (eGFR_Cys_ fully – aHR 1.47 95% CI 1.35–1.6; eGFR_Cr-Cys_ – aHR 1.36 95% CI 1.22–1.51). However, lower eGFR_Cr_ at any value was not seen to be associated with increased hazards of major bleeding compared to the reference group. In men, lower eGFR_Cr_, eGFR_Cys_ and eGFR_Cr-Cys_ were all associated with increased hazards of major bleeding, with a smaller discrepancy between between the three measures for those with eGFR > 45–60 mL/min/1.73 m^2^ (eGFR_Cr_ – aHR: 1.22, 95% CI: 1.1–1.36; eGFR_Cys_ –aHR: 1.41, 95% CI: 1.30–1.52; eGFR_Cr-Cys_ – aHR: 1.38, 95% CI: 1.25–1.52; Figure 2 and Supplementary Tables S7 and S8).

For all-cause mortality, eGFR_Cys_ and eGFR_Cr-Cys_ associated much more strongly with the outcome than eGFR_Cr_ in both men and women (Figure 2 and Supplemental Tables S9 and S10). There was no evidence of statistical interaction between sex and eGFR_Cr_, eGFR_Cys_ or eGFR_Cr-Cys_ for all-cause mortality (Table 3).

### Subpopulation with atrial fibrillation/flutter

Among 6531 participants who had AF at baseline, 1943 (29.8%) were women and 4588 (70.2%). Participants in the AF subpopulation were older compared to the overall group, with lower eGFR (all measures) and greater prevalence of cardiometabolic disease (including hypertension, diabetes, cardiovascular disease and heart failure; Supplementary Table S11). In men, over median follow-up of 11.2 (IQR 10.4–12.0) years, there were 177 ischaemic stroke and 568 major bleeding events. In women, over median follow-up 11.4 (IQR 10.6–12.1) years, there were 72 ischaemic strokes and 258 major bleeding events. For an equivalent CHADS-VASC score, prescription of a statin, an antiplatelet or an anticoagulant was less common in women compared to men (Supplemental Table S12).

In Cox proportional hazards models, lower eGFR (all measures) was associated with higher hazards of ischaemic stroke, major bleeding and a trend towards an association with haemorrhagic stroke in men, as seen in the whole population (Supplemental Figure S2). There was a similar trend in women with AF for major bleeding, but no discernible association between lower eGFR (all measures) and ischaemic or haemorrhagic stroke compared to women with eGFR > 90–105 mL/min/1.73 m^2^. For these outcomes, confidence intervals were wide reflecting very low numbers of events (Supplemental Figure S2).

### Risk discrimination of ischaemic stroke

**Figure 3.**
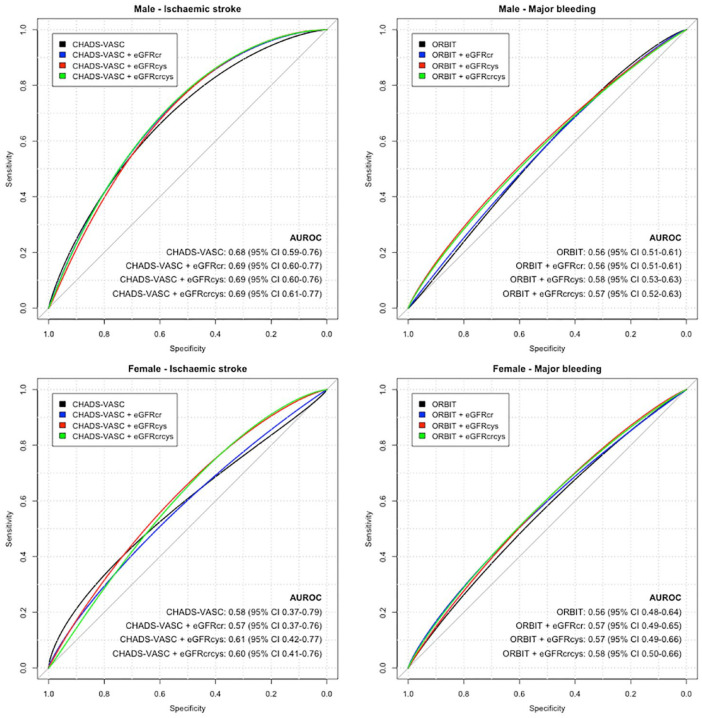
Area under receiver operating curves (AUROC) for risk discrimination of ischaemic stroke and major bleeding. CHADS-VASC: history of congestive heart failure, hypertension, diabetes, vascular disease, stroke/transient ischaemic attack or thromboembolism and age. ORBIT: age, history of major gastrointestinal or intracranial bleeding, treatment with an antiplatelet or anticoagulant, haemoglobin and haematocrit. Analyses were stratified by sex, for male participants (top) and female participants (bottom).

In men with AF at baseline, risk discrimination for ischaemic stroke (on top of variables included within the CHADS-VASC score – AUROC: 0.68, 95% CI: 0.59–0.76) showed a very small increase in AUROC after addition of eGFR_Cr_, eGFR_Cys_ and eGFR_Cr-Cys_ (AUROC for all: 0.69; Figure 3). In women with AF at baseline, CHADS-VASC both before and after addition of eGFR_Cr_, eGFR_Cys_ and eGFR_Cr-Cys_ showed poor discrimination for ischaemic stroke; the best AUROC was seen with CHADS-VASC plus eGFR_Cr-Cys_ (AUROC: 0.60, 95% CI: 0.41–0.76), but confidence intervals crossed 0.5, indicating lack of discriminative ability.

### Risk discrimination of major bleeding

In men with AF at baseline, the discriminative performance for the base model was moderate (ORBIT score – AUROC: 0.56, 95% CI: 0.51–0.61), and improved with addition of both eGFR_Cys_ (AUROC: 0.58, 95% CI: 0.53–0.63) and eGFR_Cr-Cys_ (AUROC: 0.57, 95% CI: 0.52–0.63). In women, the ORBIT score (AUROC: 0.56 95% CI: 0.48–0.64) plus eGFR_Cr-Cys_ (AUROC: 0.58 95% CI: 0.50–0.66) somewhat improved risk discrimination for major bleeding, but with suboptimal discriminatory ability overall.

## Discussion

In this cohort of volunteer participants in the UK Biobank, women had lower incidence of ischaemic and haemorrhagic stroke, major bleeding and had longer survival than men. Lower eGFR_Cys_ and eGFR_Cr-Cys_ were more strongly associated with hazards of ischaemic stroke, major bleeding and all-cause mortality than lower eGFR_Cr_ in both men and women, but this effect was more pronounced in women. Enhanced measurement of cystatin C may improve risk stratification for ischaemic stroke, major bleeding and clinical treatment decisions in a general population setting, but particularly for women.

This study is consistent with the published literature: compared with eGFR_Cr_ – the marker of kidney function which is most widely used in clinical practice – eGFR_Cys_ and eGFR_Cr-Cys_ are more strongly associated with composite cardiovascular outcomes (including myocardial infarction, stroke and cardiovascular death).^11–14^ Kidney function measures are not routinely incorporated into risk prediction tools for composite cardiovascular disease (except QRISK3, where eGFR < 60 mL/min/1.73 m^2^ is included as a binary measure).^6,8,23^ Previously, we have shown that eGFR_Cys_ improves risk discrimination for composite cardiovascular disease compared to risk calculators used in clinical practice, and across clinically meaningful thresholds (i.e., to recommend prescription of a statin) both as a continuous measure^11^ and across the threshold for diagnosis of moderate CKD.^13^ Here, we show that eGFR_Cys_ particularly can improve risk stratification for ischaemic stroke. Given the strong associations seen between CKD and risk factors for cardioembolic disease (including heart failure and propensity to arrhythmia^4^), eGFR_Cys_ or eGFR_Cr-Cys_ may be particularly helpful in stratifying risk of cardioembolic stroke, though we do not have sufficiently detailed information on stroke subtypes to confirm or refute this hypothesis in UK Biobank. Here, and in a previous study,^24^ the addition of eGFR_Cr_ made little difference to risk prediction of ischaemic stroke. Previous studies also report serum cystatin C (not incorporated into eGFR) to be associated with ischaemic stroke,^25,26^ though raw values are more difficult to interpret without acknowledging the influence of kidney function on the concentration in serum. Cystatin C-based eGFR may be useful for stratification of primary prevention decisions in stroke where stroke subtypes are not currently considered.

We identified weak associations between kidney function markers and haemorrhagic stroke in men (but not in women), and these were substantially less pronounced than for ischaemic stroke, with various potential explanations. First, though advanced CKD is associated with major bleeding due to platelet dysfunction and anticoagulation use during dialysis,^3^ the UK Biobank population has only a very small proportion (<1%) of people with advanced CKD (eGFR < 45 mL/min/1.73 m^2^): above this threshold, CKD-specific effects on bleeding risk may be less important. Second, CKD is a marker of severity for other risk factors for haemorrhagic stroke,^3^ and may not itself be associated with substantial increase in haemorrhagic stroke risk. Third, there were 80% fewer haemorrhagic than ischaemic strokes: the lack of strong associations may reflect reduced power for this outcome.

We have shown that risk discrimination using CHADS-VASC for ischaemic stroke is similar in male UK Biobank participants compared with its performance in other cohorts,^27^ and was improved by the addition of all of eGFR_Cr_, eGFR_Cr-Cys_ and eGFR_Cys_ (greatest improvement). Risk discrimination for major bleeding was also improved by addition of eGFR_Cys_ and eGFR_Cr-Cys_ in men, though the base model demonstrated lower discrimination than in the original development and validation cohorts.^22^ However, the base CHADS-VASC and ORBIT models had poor discrimination for ischaemic stroke and major bleeding respectively in women in UK Biobank, with both models performing little better than a coin toss even after the inclusion of kidney function measures. While the differential performance of stroke risk prediction tools may be a reflection of inadequate power in women in this study (1943 women had AF, of whom 72 had a stroke during follow-up), female sex has been shown to be a risk modifier rather than risk factor for stroke in patients with AF, such that risk of stroke is accentuated in women compared with men with two more risk factors for stroke.^28^ Sex-specific risk factors for stroke may also account for this discrepancy.

Cardiometabolic diseases (such a hypertension, diabetes, dyslipidaemia) are important risk factors for stroke. However, there are additional male- and female-specific risk factors for stroke that are not accounted for in risk prediction tools. In this population, men had substantially greater burden of cardiometabolic disease than women, which may explain the better baseline performance of the CHADS-VASC risk score in male participants. Non-cardiometabolic risk factors for stroke in men include medical androgen deprivation therapy (e.g., bicalutamide) and/or erectile dysfunction, though the latter may also be a reflection of microvascular dysfunction in association with cardiometabolic disease.^29,30^ By comparison, women are exposed to a lifetime of hormone fluctuations. In particular, pregnancy and hormone-related risk factors (including gestational hypertension, pre-term delivery, stillbirth, earlier age of menopause and oophorectomy) confer substantial additional risk of stroke.^31,32^ Women with kidney disease are subject to additional hormone dysregulation, which may further enhance risk of stroke beyond traditional risk factors.^33^ This may explain the larger relative increase in hazards of ischaemic stroke in women compared with men, particularly in those with mild CKD.

There are also differences in kidney function – and the measures used to estimate kidney function – between men and women. Compared with men, women typically have lower measured kidney function^34,35^ and slower change in kidney function with increasing age.^34^ However, the differential improvement in risk stratification between eGFR_Cr_, eGFR_Cys_ and eGFR_Cr-Cys_ are likely explained by the non-GFR determinants of both creatinine and cystatin C. Creatinine is released from muscle, and measured in serum (at constant GFR) is most strongly influenced by muscle mass and activity.^36^ At extremes of muscle mass, serum creatinine may over- or under-estimate kidney function. By comparison, cystatin C is released at a constant rate in health and is freely filtered at the glomerulus; however, cystatin C levels may be stimulated by inflammation, steroid use and are commonly elevated in cardiometabolic disease such as diabetes and obesity. With increasing age, cardiometabolic ill-health and frailty, there becomes a widening discrepancy between eGFR_Cr_ and eGFR_Cys_.^13,37,38^ Owing in part to lower muscle mass,^39^ lower loss of muscle mass with increasing age^40^ and lower burden of cardiometabolic disease,^41,42^ women may have greater discordance between eGFR_Cr_ and eGFR_Cys_ than men. Though both eGFR_Cr_ and eGFR_Cys_ may be unreliable markers of kidney function at extremes of body composition or ill health, eGFR_Cr_ more commonly overestimates kidney function in women, and therefore underestimates risk associated with CKD and reduces their eligibility for risk-reduction strategies.^43,44^ The added value of using cystatin C-based (in preference to creatinine-based) measures for risk stratification for ischaemic stroke and major bleeding is greater in women.

### Strengths and limitations

The strengths of this study are in the extensive phenotyping of a large participant cohort, over long period of follow-up with excellent capture of relevant outcome data using linked records. However, we acknowledge some limitations. First, we included only people who had not had a previous ischaemic stroke or major bleeding event. Although this is typical for an analysis of this type, kidney function is a risk factor for ischaemic stroke and previous episodes of ischaemic stroke are known to increase the risk of future events: it is possible that the added value of eGFR_Cys_ for risk discrimination would be reduced in those with established cerebrovascular disease. Second, there are limitations relating to the UK Biobank algorithm for capturing ischaemic stroke events: there is no information on stroke severity; the algorithm captures only the first event (fatal or non-fatal) during the follow-up period, and we cannot comment on the frequency of recurrent stroke disease. Furthermore, the algorithm uses ICD-9 or ICD-10 codes, and does not allow stroke subtype stratification according to aetiological classifications such as TOAST (Trial of ORG 10172 in Acute Stroke Treatment) or ASCOD (Atherosclerosis; Small-vessel disease; Cardiac pathology; Other; Dissection). Third, the UK Biobank population was limited to participants aged 40–73 years at the start of follow-up: we cannot guarantee generalisability of these findings to younger or older age groups. Fourth, in keeping with many epidemiological studies of this type, there was only a single measure of kidney function captured at baseline, and we cannot account for variation in kidney function over time. Fifth, no participant in UK Biobank was prescribed direct oral anticoagulants or newer antiplatelets (including ticagrelor), as the recruitment period ran prior to the routine introduction of these medications into clinical practice. Sixth, this population had relatively preserved kidney function: 95% had eGFR_Cr_ > 60 and 99% had eGFR_Cr_ > 45. It is unlikely that these findings can be generalised to people with more advanced kidney disease. Similarly, we had access to only a single baseline measure of kidney function, thus we cannot comment on the potential impact of kidney disease progression. Furthermore, this population had very low levels of albuminuria, which is itself a risk factor for kidney disease progression and stroke.^2^ Nevertheless, the results are likely to be applicable to the level of kidney function routinely managed in the primary care or non-specialist setting. Last, UK Biobank is a volunteer cohort that is relatively health compared to the general population, with lower absolute risks of various outcomes (including stroke).^45,46^ However, previous analyses indicate that the hazard ratios should still be applicable to the general UK population.^45,46^

## Conclusion

In conclusion, we show that compared with eGFR_Cr_, eGFR_Cys_ and eGFR_Cr-Cys_ more sensitively detect and more strongly associate with future risk of ischaemic stroke and major bleeding in the UK Biobank cohort. The discrepancy between cystatin C- and creatinine-based eGFR for detecting risk of ischaemic stroke was greater in women than in men. Enhanced measurement of cystatin C may improve risk stratification and clinical treatment decisions for ischaemic stroke and major bleeding in a general population setting, particularly for women.

## Supplemental Material

sj-docx-1-eso-10.1177_23969873231173282 – Supplemental material for Sex differences in associations between creatinine and cystatin C-based kidney function measures with stroke and major bleeding Click here for additional data file. Supplemental material, sj-docx-1-eso-10.1177_23969873231173282 for Sex differences in associations between creatinine and cystatin C-based kidney function measures with stroke and major bleeding by Jennifer Susan Lees, Nicole L De La Mata, Michael K Sullivan, Melanie L Wyld, Brenda M Rosales, Rachel Cutting, James Alan Hedley, Elaine Rutherford, Patrick Barry Mark and Angela C Webster in European Stroke Journal
