# Supplementary material for: Sex differences in associations between creatinine and cystatin C-based kidney function measures with stroke and major bleeding
Source: Eur Stroke J. 2023 May 12;8(3):756–68. doi: 10.1177/23969873231173282 (PMC10465308; doi:10.1177/23969873231173282)
Supplement: sj-docx-1-eso-10.1177_23969873231173282 – Supplemental material for Sex differences in associations between creatinine and cystatin C-based kidney function measures with stroke and major bleeding [file sj-docx-1-eso-10.1177_23969873231173282.docx]

# **Supplementary Data**

**Table of contents**

Table S1: Number of events by estimated glomerular filtration rate based on serum cystatin C (eGFRcys) category.

Table S2: Number of events by estimated glomerular filtration rate based on serum creatinine and cystatin C (eGFRcrcys) category.

Table S3: Cause-specific Cox proportional hazards model outputs in male participants for ischaemic stroke outcome

Table S4: Cause-specific Cox proportional hazards model outputs in female participants for ischaemic stroke outcome

Table S5: Cause-specific Cox proportional hazards model outputs in male participants for haemorrhagic stroke outcome

Table S6: Cause-specific Cox proportional hazards model outputs in female participants for haemorrhagic stroke outcome

Table S7: Cause-specific Cox proportional hazards model outputs in male participants for major bleeding outcome

Table S8: Cause-specific Cox proportional hazards model outputs in female participants for major bleeding outcome

Table S9: Cause-specific Cox proportional hazards model outputs in male participants for all-cause mortality

Table S10: Cause-specific Cox proportional hazards model outputs in female participants for all-cause mortality

Table S11: Baseline characteristics in the subgroup with atrial fibrillation/flutter at baseline

Table S12: Proportion of people with pre-existing atrial fibrillation/flutter prescribed preventative medications by sex and CHADS-VASC score.

Figure S1: Density plot showing the differences in eGFRcr, eGFRcys and eGFRcrcys by sex in the UK Biobank population

Figure S2: Forest plots displaying cause-specific hazard ratio and 95% confidence intervals (95% CI) for ischaemic stroke, haemorrhagic stroke, major bleeding and all-cause mortality in a sub-population of participants with atrial fibrillation/flutter at baseline.

## Table S1

| **Sex** | **eGFRcys category**  **(mL/min/1.73m^2^)** | **N** | **Ischaemic stroke (N=)** | **Haemorrhagic strokes (N=)** | **Major bleed (N=)** | **All-cause mortality (N=)** |
| --- | --- | --- | --- | --- | --- | --- |
| **Male** | >105 | 25,289 | 140 | 33 | 1,457 | 1,239 |
|  | >90-105 | 95,467 | 953 | 174 | 5,720 | 7,003 |
|  | >75-90 | 60,675 | 724 | 150 | 3,882 | 5,321 |
|  | >60-75 | 20,757 | 334 | 66 | 1,561 | 2,427 |
|  | >45-60 | 3,660 | 115 | 23 | 387 | 777 |
|  | >30-45 | 641 | 31 | 5 | 102 | 242 |
|  | <=30 | 170 | 6 | 2 | 30 | 74 |
| **Female** | >105 | 32,494 | 78 | 22 | 1,484 | 755 |
|  | >90-105 | 115,472 | 558 | 154 | 5,967 | 5,222 |
|  | >75-90 | 67,433 | 432 | 91 | 3,491 | 3,351 |
|  | >60-75 | 25,522 | 254 | 59 | 1,551 | 1,738 |
|  | >45-60 | 4,572 | 65 | 15 | 322 | 498 |
|  | >30-45 | 608 | 10 | 1 | 57 | 144 |
|  | <=30 | 119 | 6 | 0 | 14 | 47 |

Number of events by estimated glomerular filtration rate based on serum creatinine (eGFRcys) category.

## Table S2

| **Sex** | **eGFRcrcys category**  **(mL/min/1.73m^2^)** | **N** | **Ischaemic stroke (N=)** | **Haemorrhagic strokes (N=)** | **Major bleed (N=)** | **All-cause mortality (N=)** |
| --- | --- | --- | --- | --- | --- | --- |
| **Male** | >105 | 25,289 | 140 | 33 | 1,457 | 1,239 |
|  | >90-105 | 95,467 | 953 | 174 | 5,720 | 7,003 |
|  | >75-90 | 60,675 | 724 | 150 | 3,882 | 5,321 |
|  | >60-75 | 20,757 | 334 | 66 | 1,561 | 2,427 |
|  | >45-60 | 3,660 | 115 | 23 | 387 | 777 |
|  | >30-45 | 6,41 | 31 | 5 | 102 | 242 |
|  | <=30 | 170 | 6 | 2 | 30 | 74 |
| **Female** | >105 | 32,494 | 78 | 22 | 1,484 | 755 |
|  | >90-105 | 115,472 | 558 | 154 | 5,967 | 5,222 |
|  | >75-90 | 67,433 | 432 | 91 | 3,491 | 3,351 |
|  | >60-75 | 25,522 | 254 | 59 | 1,551 | 1,738 |
|  | >45-60 | 4,572 | 65 | 15 | 322 | 498 |
|  | >30-45 | 608 | 10 | 1 | 57 | 144 |
|  | <=30 | 119 | 6 | 0 | 14 | 47 |

Number of events by estimated glomerular filtration rate based on serum creatinine (eGFRcrcys) category.

## Table S3

Cause-specific Cox proportional hazards model outputs in male participants for ischaemic stroke outcome

|  |  | **eGFRcr** |  | **eGFRcys** |  | **eGFRcrcys** |  |
| --- | --- | --- | --- | --- | --- | --- | --- |
| **Model** | **eGFR category**  **(mL/min/1.73m^2^)** | **HR (95% CI)** | **P value** | **HR (95% CI)** | **P value** | **HR (95% CI)** | **P value** |
| Model 1 | >105 | 1.25 (1.03-1.51) | 0.025 | 0.85 (0.71-1.02) | 0.089 | 0.94 (0.77-1.15) | 0.55 |
|  | >90-105 | 1 (Ref) | NA | 1 (Ref) | NA | 1 (Ref) | NA |
|  | >75-90 | 0.94 (0.85-1.04) | 0.226 | 1.09 (0.97-1.22) | 0.163 | 1.15 (1.04-1.28) | 0.008 |
|  | >60-75 | 1.06 (0.93-1.2) | 0.389 | 1.34 (1.18-1.52) | <0.001 | 1.22 (1.07-1.39) | 0.003 |
|  | >45-60 | 1.64 (1.34-2) | <0.001 | 1.6 (1.35-1.9) | <0.001 | 1.98 (1.63-2.4) | <0.001 |
|  | >30-45 | 2.05 (1.43-2.96) | <0.001 | 2.5 (1.92-3.25) | <0.001 | 2.25 (1.61-3.13) | <0.001 |
|  | <=30 | 2.01 (0.9-4.5) | 0.089 | 2.71 (1.58-4.65) | <0.001 | 3.12 (1.71-5.7) | <0.001 |
| Model 2 | >105 | 1.24 (1.03-1.51) | 0.026 | 0.85 (0.71-1.02) | 0.088 | 0.94 (0.77-1.15) | 0.546 |
|  | >90-105 | 1 (Ref) | NA | 1 (Ref) | NA | 1 (Ref) | NA |
|  | >75-90 | 0.94 (0.85-1.04) | 0.231 | 1.09 (0.97-1.22) | 0.16 | 1.15 (1.04-1.28) | 0.008 |
|  | >60-75 | 1.06 (0.93-1.2) | 0.38 | 1.34 (1.18-1.52) | <0.001 | 1.22 (1.07-1.39) | 0.003 |
|  | >45-60 | 1.63 (1.34-1.99) | <0.001 | 1.6 (1.34-1.9) | <0.001 | 1.98 (1.63-2.39) | <0.001 |
|  | >30-45 | 2.06 (1.43-2.97) | <0.001 | 2.5 (1.92-3.26) | <0.001 | 2.25 (1.62-3.14) | <0.001 |
|  | <=30 | 2.01 (0.9-4.5) | 0.089 | 2.74 (1.6-4.7) | <0.001 | 3.15 (1.72-5.75) | <0.001 |
| Model 3 | >105 | 1.23 (1.02-1.5) | 0.034 | 0.85 (0.71-1.02) | 0.088 | 0.94 (0.77-1.14) | 0.52 |
|  | >90-105 | 1 (Ref) | NA | 1 (Ref) | NA | 1 (Ref) | NA |
|  | >75-90 | 0.94 (0.85-1.04) | 0.243 | 1.08 (0.96-1.21) | 0.202 | 1.14 (1.03-1.27) | 0.012 |
|  | >60-75 | 1.06 (0.93-1.2) | 0.405 | 1.32 (1.16-1.5) | <0.001 | 1.2 (1.05-1.37) | 0.007 |
|  | >45-60 | 1.63 (1.33-1.99) | <0.001 | 1.57 (1.32-1.87) | <0.001 | 1.94 (1.6-2.35) | <0.001 |
|  | >30-45 | 1.97 (1.36-2.86) | <0.001 | 2.44 (1.87-3.18) | <0.001 | 2.17 (1.55-3.04) | <0.001 |
|  | <=30 | 1.97 (0.88-4.42) | 0.099 | 2.72 (1.58-4.67) | <0.001 | 3.07 (1.68-5.61) | <0.001 |

eGFR: estimated glomerular filtration rate. Model 1: for accepted age, smoking, systolic and diastolic blood pressure, medications for blood pressure or cholesterol, baseline total, LDL and HDL cholesterol, pre-existing heart failure, atrial fibrillation/flutter or other atherosclerotic cardiovascular disease (including myocardial infarction and peripheral vascular disease). Model 2 was adjusted for variables included in Model 1, plus prescription of an anticoagulant (warfarin or low-molecular weight heparin) or an antiplatelet agent (aspirin, clopidogrel, prasugrel or dipyridamole) at baseline. Model 3 was adjusted for variables included in Model 2, plus ethnicity, body mass index and hip to waist ratio.

## Table S4

Cause-specific Cox proportional hazards model outputs in female participants for ischaemic stroke outcome

|  |  | **eGFRcr** |  | **eGFRcys** |  | **eGFRcrcys** |  |
| --- | --- | --- | --- | --- | --- | --- | --- |
| **Model** | **eGFR category**  **(mL/min/1.73m^2^)** | **HR (95% CI)** | **P value** | **HR (95% CI)** | **P value** | **HR (95% CI)** | **P value** |
| Model 1 | >105 | 1.39 (1.07-1.8) | 0.012 | 0.91 (0.68-1.22) | 0.527 | 1.1 (0.86-1.4) | 0.471 |
|  | >90-105 | 1 (Ref) | NA | 1 (Ref) | NA | 1 (Ref) | NA |
|  | >75-90 | 1.1 (0.97-1.25) | 0.147 | 1.09 (0.93-1.27) | 0.283 | 1.2 (1.04-1.38) | 0.012 |
|  | >60-75 | 1.34 (1.15-1.56) | <0.001 | 1.56 (1.33-1.83) | <0.001 | 1.72 (1.47-2.01) | <0.001 |
|  | >45-60 | 1.42 (1.09-1.84) | 0.009 | 2.18 (1.79-2.67) | <0.001 | 2.02 (1.58-2.56) | <0.001 |
|  | >30-45 | 1.26 (0.67-2.37) | 0.469 | 2.89 (2.08-4.01) | <0.001 | 2.7 (1.74-4.17) | <0.001 |
|  | <=30 | 4.57 (2.02-10.35) | <0.001 | 2.94 (1.37-6.32) | 0.006 | 3.06 (1.25-7.49) | 0.014 |
| Model 2 | >105 | 1.39 (1.07-1.8) | 0.013 | 0.91 (0.68-1.22) | 0.526 | 1.09 (0.85-1.4) | 0.476 |
|  | >90-105 | 1 (Ref) | NA | 1 (Ref) | NA | 1 (Ref) | NA |
|  | >75-90 | 1.1 (0.97-1.25) | 0.147 | 1.09 (0.93-1.26) | 0.287 | 1.2 (1.04-1.38) | 0.012 |
|  | >60-75 | 1.34 (1.15-1.56) | <0.001 | 1.56 (1.33-1.83) | <0.001 | 1.72 (1.46-2.01) | <0.001 |
|  | >45-60 | 1.41 (1.08-1.83) | 0.011 | 2.18 (1.78-2.66) | <0.001 | 2 (1.57-2.55) | <0.001 |
|  | >30-45 | 1.26 (0.67-2.37) | 0.472 | 2.87 (2.07-3.98) | <0.001 | 2.68 (1.73-4.16) | <0.001 |
|  | <=30 | 4.52 (2-10.24) | <0.001 | 2.95 (1.38-6.32) | 0.005 | 3.06 (1.25-7.48) | 0.014 |
| Model 3 | >105 | 1.36 (1.04-1.76) | 0.023 | 0.89 (0.66-1.21) | 0.465 | 1.1 (0.86-1.41) | 0.464 |
|  | >90-105 | 1 (Ref) | NA | 1 (Ref) | NA | 1 (Ref) | NA |
|  | >75-90 | 1.08 (0.95-1.23) | 0.217 | 1.08 (0.93-1.26) | 0.319 | 1.19 (1.03-1.37) | 0.017 |
|  | >60-75 | 1.31 (1.12-1.52) | 0.001 | 1.54 (1.31-1.81) | <0.001 | 1.68 (1.43-1.97) | <0.001 |
|  | >45-60 | 1.37 (1.05-1.78) | 0.019 | 2.13 (1.73-2.62) | <0.001 | 1.92 (1.5-2.45) | <0.001 |
|  | >30-45 | 1.22 (0.65-2.3) | 0.535 | 2.78 (1.99-3.89) | <0.001 | 2.62 (1.69-4.06) | <0.001 |
|  | <=30 | 4.29 (1.89-9.72) | <0.001 | 2.9 (1.35-6.24) | 0.006 | 2.97 (1.21-7.28) | 0.017 |

eGFR: estimated glomerular filtration rate. Model 1: for accepted age, smoking, systolic and diastolic blood pressure, medications for blood pressure or cholesterol, baseline total, LDL and HDL cholesterol, pre-existing heart failure, atrial fibrillation/flutter or other atherosclerotic cardiovascular disease (including myocardial infarction and peripheral vascular disease). Model 2 was adjusted for variables included in Model 1, plus prescription of an anticoagulant (warfarin or low-molecular weight heparin) or an antiplatelet agent (aspirin, clopidogrel, prasugrel or dipyridamole) at baseline. Model 3 was adjusted for variables included in Model 2, plus ethnicity, body mass index and hip to waist ratio.

## Table S5

Cause-specific Cox proportional hazards model outputs in male participants for haemorrhagic stroke outcome

|  |  | **eGFRcr** |  | **eGFRcys** |  | **eGFRcrcys** |  |
| --- | --- | --- | --- | --- | --- | --- | --- |
| **Model** | **eGFR category**  **(mL/min/1.73m^2^)** | **HR (95% CI)** | **P value** | **HR (95% CI)** | **P value** | **HR (95% CI)** | **P value** |
| Model 1 | >105 | 1.77 (1.18-2.65) | 0.006 | 0.78 (0.53-1.14) | 0.197 | 0.97 (0.64-1.47) | 0.889 |
|  | >90-105 | 1 (Ref) | NA | 1 (Ref) | NA | 1 (Ref) | NA |
|  | >75-90 | 1.04 (0.83-1.3) | 0.71 | 0.85 (0.67-1.1) | 0.22 | 0.93 (0.74-1.17) | 0.523 |
|  | >60-75 | 1.16 (0.87-1.55) | 0.306 | 1.15 (0.88-1.51) | 0.298 | 1.26 (0.95-1.67) | 0.107 |
|  | >45-60 | 2.01 (1.29-3.13) | 0.002 | 1.32 (0.89-1.97) | 0.168 | 1.89 (1.21-2.95) | 0.005 |
|  | >30-45 | 2.43 (0.99-5.97) | 0.053 | 2.55 (1.39-4.67) | 0.003 | 2.53 (1.17-5.47) | 0.019 |
|  | <=30 | 3.97 (0.97-16.23) | 0.055 | 4.58 (1.66-12.63) | 0.003 | 3.11 (0.76-12.74) | 0.115 |
| Model 2 | >105 | 1.71 (1.14-2.58) | 0.009 | 0.77 (0.52-1.14) | 0.188 | 0.96 (0.64-1.45) | 0.857 |
|  | >90-105 | 1 (Ref) | NA | 1 (Ref) | NA | 1 (Ref) | NA |
|  | >75-90 | 1.04 (0.83-1.3) | 0.742 | 0.85 (0.66-1.09) | 0.208 | 0.92 (0.73-1.16) | 0.492 |
|  | >60-75 | 1.13 (0.84-1.51) | 0.415 | 1.12 (0.85-1.46) | 0.423 | 1.2 (0.91-1.59) | 0.195 |
|  | >45-60 | 1.82 (1.17-2.84) | 0.008 | 1.21 (0.81-1.8) | 0.359 | 1.67 (1.07-2.62) | 0.024 |
|  | >30-45 | 2.16 (0.88-5.3) | 0.094 | 2.17 (1.18-3.99) | 0.013 | 2.17 (1-4.7) | 0.05 |
|  | <=30 | 3.61 (0.88-14.78) | 0.074 | 4.17 (1.51-11.51) | 0.006 | 2.83 (0.69-11.6) | 0.148 |
| Model 3 | >105 | 1.73 (1.14-2.6) | 0.009 | 0.77 (0.52-1.13) | 0.181 | 0.96 (0.63-1.45) | 0.843 |
|  | >90-105 | 1 (Ref) | NA | 1 (Ref) | NA | 1 (Ref) | NA |
|  | >75-90 | 1.04 (0.83-1.3) | 0.751 | 0.85 (0.66-1.09) | 0.207 | 0.92 (0.73-1.16) | 0.469 |
|  | >60-75 | 1.12 (0.84-1.5) | 0.44 | 1.11 (0.84-1.46) | 0.458 | 1.19 (0.9-1.58) | 0.23 |
|  | >45-60 | 1.79 (1.15-2.8) | 0.011 | 1.19 (0.79-1.79) | 0.397 | 1.64 (1.04-2.58) | 0.032 |
|  | >30-45 | 2.11 (0.86-5.2) | 0.104 | 2.14 (1.15-3.96) | 0.016 | 2.11 (0.97-4.6) | 0.06 |
|  | <=30 | 3.49 (0.85-14.29) | 0.082 | 4.09 (1.48-11.33) | 0.007 | 2.71 (0.66-11.13) | 0.167 |

eGFR: estimated glomerular filtration rate. Model 1 was adjusted for age, history of or medication for hypertension, systolic or diastolic blood pressure, haemoglobin and haematocrit. Model 2 was adjusted for variables included in Model 1, plus prescription of an anticoagulant (warfarin or low-molecular weight heparin) or an antiplatelet agent (aspirin, clopidogrel, prasugrel or dipyridamole) at baseline. Model 3 was adjusted for variables included in Model 2, plus ethnicity, body mass index and hip to waist ratio.

## Table S6

Cause-specific Cox proportional hazards model outputs in female participants for haemorrhagic stroke outcome

|  |  | **eGFRcr** |  | **eGFRcys** |  | **eGFRcrcys** |  |
| --- | --- | --- | --- | --- | --- | --- | --- |
| **Model** | **eGFR category**  **(mL/min/1.73m^2^)** | **HR (95% CI)** | **P value** | **HR (95% CI)** | **P value** | **HR (95% CI)** | **P value** |
|  |  |  |  |  |  |  |  |
| Model 1 | >105 | 1.49 (0.91-2.44) | 0.113 | 1.28 (0.8-2.05) | 0.307 | 1.33 (0.88-2) | 0.172 |
|  | >90-105 | 1 (Ref) | NA | 1 (Ref) | NA | 1 (Ref) | NA |
|  | >75-90 | 0.83 (0.64-1.08) | 0.17 | 0.79 (0.59-1.05) | 0.099 | 0.72 (0.55-0.94) | 0.015 |
|  | >60-75 | 1.17 (0.86-1.58) | 0.326 | 1.06 (0.78-1.43) | 0.712 | 1.02 (0.75-1.4) | 0.891 |
|  | >45-60 | 1.32 (0.77-2.27) | 0.306 | 1.24 (0.81-1.89) | 0.332 | 1.48 (0.91-2.41) | 0.119 |
|  | >30-45 | 0.57 (0.08-4.08) | 0.574 | 1.2 (0.48-2.98) | 0.694 | 1.32 (0.42-4.2) | 0.638 |
|  | <=30 | 0 (0-Inf) | 0.986 | 0 (0-Inf) | 0.987 | 0 (0-Inf) | 0.99 |
| Model 2 | >105 | 1.47 (0.9-2.41) | 0.126 | 1.27 (0.79-2.04) | 0.319 | 1.32 (0.88-1.98) | 0.182 |
|  | >90-105 | 1 (Ref) | NA | 1 (Ref) | NA | 1 (Ref) | NA |
|  | >75-90 | 0.83 (0.64-1.08) | 0.162 | 0.78 (0.59-1.04) | 0.093 | 0.71 (0.54-0.93) | 0.013 |
|  | >60-75 | 1.14 (0.84-1.55) | 0.409 | 1.04 (0.77-1.4) | 0.809 | 1 (0.73-1.37) | 0.992 |
|  | >45-60 | 1.26 (0.74-2.17) | 0.399 | 1.17 (0.76-1.8) | 0.473 | 1.38 (0.84-2.25) | 0.203 |
|  | >30-45 | 0.52 (0.07-3.73) | 0.515 | 1.07 (0.43-2.66) | 0.89 | 1.17 (0.37-3.73) | 0.792 |
|  | <=30 | 0 (0-Inf) | 0.987 | 0 (0-Inf) | 0.989 | 0 (0-Inf) | 0.986 |
| Model 3 | >105 | 1.43 (0.86-2.38) | 0.173 | 1.21 (0.75-1.95) | 0.427 | 1.28 (0.85-1.93) | 0.242 |
|  | >90-105 | 1 (Ref) | NA | 1 (Ref) | NA | 1 (Ref) | NA |
|  | >75-90 | 0.86 (0.66-1.12) | 0.252 | 0.84 (0.63-1.12) | 0.245 | 0.77 (0.58-1.01) | 0.058 |
|  | >60-75 | 1.2 (0.88-1.63) | 0.25 | 1.19 (0.87-1.62) | 0.283 | 1.13 (0.82-1.57) | 0.444 |
|  | >45-60 | 1.36 (0.79-2.34) | 0.263 | 1.44 (0.92-2.24) | 0.109 | 1.63 (0.98-2.68) | 0.057 |
|  | >30-45 | 0.56 (0.08-4.02) | 0.564 | 1.38 (0.55-3.48) | 0.491 | 1.41 (0.44-4.52) | 0.563 |
|  | <=30 | 0 (0-Inf) | 0.995 | 0 (0-Inf) | 0.993 | 0 (0-Inf) | 0.994 |

eGFR: estimated glomerular filtration rate. Model 1 was adjusted for age, history of or medication for hypertension, systolic or diastolic blood pressure, haemoglobin and haematocrit. Model 2 was adjusted for variables included in Model 1, plus prescription of an anticoagulant (warfarin or low-molecular weight heparin) or an antiplatelet agent (aspirin, clopidogrel, prasugrel or dipyridamole) at baseline. Model 3 was adjusted for variables included in Model 2, plus ethnicity, body mass index and hip to waist ratio.

## Table S7

Cause-specific Cox proportional hazards model outputs in male participants for major bleeding outcome

|  |  | **eGFRcr** |  | **eGFRcys** |  | **eGFRcrcys** |  |
| --- | --- | --- | --- | --- | --- | --- | --- |
| **Model** | **eGFR category**  **(mL/min/1.73m^2^)** | **HR (95% CI)** | **P value** | **HR (95% CI)** | **P value** | **HR (95% CI)** | **P value** |
| Model 1 | >105 | 1.34 (1.26-1.42) | <0.001 | 0.92 (0.87-0.98) | 0.009 | 1 (0.94-1.07) | 0.949 |
|  | >90-105 | 1 (Ref) | NA | 1 (Ref) | NA | 1 (Ref) | NA |
|  | >75-90 | 0.97 (0.93-1.01) | 0.111 | 1.03 (0.98-1.08) | 0.227 | 1.04 (0.99-1.08) | 0.107 |
|  | >60-75 | 1.06 (1-1.12) | 0.049 | 1.22 (1.16-1.29) | <0.001 | 1.2 (1.14-1.27) | <0.001 |
|  | >45-60 | 1.36 (1.22-1.51) | <0.001 | 1.6 (1.48-1.73) | <0.001 | 1.6 (1.45-1.77) | <0.001 |
|  | >30-45 | 1.94 (1.59-2.36) | <0.001 | 2.03 (1.77-2.34) | <0.001 | 2.07 (1.74-2.46) | <0.001 |
|  | <=30 | 2.11 (1.47-3.03) | <0.001 | 2.62 (2.02-3.39) | <0.001 | 2.23 (1.62-3.06) | <0.001 |
| Model 2 | >105 | 1.32 (1.23-1.4) | <0.001 | 0.92 (0.87-0.98) | 0.007 | 1 (0.94-1.06) | 0.902 |
|  | >90-105 | 1 (Ref) | NA | 1 (Ref) | NA | 1 (Ref) | NA |
|  | >75-90 | 0.96 (0.92-1) | 0.08 | 1.02 (0.98-1.07) | 0.303 | 1.03 (0.99-1.07) | 0.173 |
|  | >60-75 | 1.04 (0.98-1.1) | 0.209 | 1.19 (1.13-1.26) | <0.001 | 1.17 (1.1-1.23) | <0.001 |
|  | >45-60 | 1.27 (1.14-1.41) | <0.001 | 1.5 (1.39-1.62) | <0.001 | 1.48 (1.34-1.63) | <0.001 |
|  | >30-45 | 1.76 (1.44-2.14) | <0.001 | 1.83 (1.59-2.11) | <0.001 | 1.85 (1.56-2.2) | <0.001 |
|  | <=30 | 1.94 (1.35-2.78) | <0.001 | 2.38 (1.83-3.08) | <0.001 | 2.03 (1.47-2.78) | <0.001 |
| Model 3 | >105 | 1.29 (1.21-1.37) | <0.001 | 0.93 (0.88-0.99) | 0.028 | 1 (0.94-1.06) | 0.969 |
|  | >90-105 | 1 (Ref) | NA | 1 (Ref) | NA | 1 (Ref) | NA |
|  | >75-90 | 0.96 (0.92-1) | 0.07 | 1.01 (0.96-1.06) | 0.744 | 1.01 (0.97-1.05) | 0.619 |
|  | >60-75 | 1.02 (0.97-1.09) | 0.413 | 1.15 (1.09-1.21) | <0.001 | 1.12 (1.06-1.19) | <0.001 |
|  | >45-60 | 1.22 (1.1-1.36) | <0.001 | 1.41 (1.3-1.52) | <0.001 | 1.38 (1.25-1.52) | <0.001 |
|  | >30-45 | 1.62 (1.32-1.98) | <0.001 | 1.66 (1.44-1.91) | <0.001 | 1.68 (1.41-2) | <0.001 |
|  | <=30 | 1.77 (1.23-2.54) | 0.002 | 2.16 (1.66-2.79) | <0.001 | 1.81 (1.32-2.49) | <0.001 |

eGFR: estimated glomerular filtration rate. Model 1 was adjusted for age, history of or medication for hypertension, systolic or diastolic blood pressure, haemoglobin and haematocrit. Model 2 was adjusted for variables included in Model 1, plus prescription of an anticoagulant (warfarin or low-molecular weight heparin) or an antiplatelet agent (aspirin, clopidogrel, prasugrel or dipyridamole) at baseline. Model 3 was adjusted for variables included in Model 2, plus ethnicity, body mass index and hip to waist ratio.

## Table S8

Cause-specific Cox proportional hazards model outputs in female participants for major bleeding outcome

|  |  | **eGFRcr** |  | **eGFRcys** |  | **eGFRcrcys** |  |
| --- | --- | --- | --- | --- | --- | --- | --- |
| **Model** | **eGFR category**  **(mL/min/1.73m^2^)** | **HR (95% CI)** | **P value** | **HR (95% CI)** | **P value** | **HR (95% CI)** | **P value** |
| Model 1 | >105 | 1.14 (1.07-1.21) | <0.001 | 0.99 (0.93-1.06) | 0.78 | 1.05 (0.99-1.11) | 0.086 |
|  | >90-105 | 1 (Ref) | NA | 1 (Ref) | NA | 1 (Ref) | NA |
|  | >75-90 | 0.96 (0.92-1) | 0.034 | 1.1 (1.05-1.15) | <0.001 | 1.04 (1-1.09) | 0.046 |
|  | >60-75 | 1.06 (1-1.12) | 0.062 | 1.25 (1.18-1.32) | <0.001 | 1.19 (1.13-1.26) | <0.001 |
|  | >45-60 | 1.13 (1.01-1.27) | 0.03 | 1.63 (1.5-1.76) | <0.001 | 1.53 (1.38-1.7) | <0.001 |
|  | >30-45 | 1.42 (1.09-1.84) | 0.009 | 1.98 (1.68-2.32) | <0.001 | 1.69 (1.35-2.13) | <0.001 |
|  | <=30 | 1.81 (1.07-3.07) | 0.026 | 2.1 (1.45-3.03) | <0.001 | 2.14 (1.39-3.29) | 0.001 |
| Model 2 | >105 | 1.12 (1.05-1.2) | <0.001 | 0.99 (0.93-1.05) | 0.696 | 1.05 (0.99-1.11) | 0.118 |
|  | >90-105 | 1 (Ref) | NA | 1 (Ref) | NA | 1 (Ref) | NA |
|  | >75-90 | 0.95 (0.91-0.99) | 0.025 | 1.09 (1.04-1.14) | <0.001 | 1.04 (1-1.09) | 0.072 |
|  | >60-75 | 1.04 (0.98-1.1) | 0.187 | 1.23 (1.16-1.29) | <0.001 | 1.17 (1.1-1.24) | <0.001 |
|  | >45-60 | 1.08 (0.97-1.21) | 0.177 | 1.55 (1.44-1.68) | <0.001 | 1.44 (1.3-1.59) | <0.001 |
|  | >30-45 | 1.29 (0.99-1.67) | 0.06 | 1.78 (1.51-2.1) | <0.001 | 1.5 (1.19-1.89) | 0.001 |
|  | <=30 | 1.65 (0.97-2.78) | 0.063 | 1.88 (1.3-2.71) | 0.001 | 1.93 (1.26-2.97) | 0.003 |
| Model 3 | >105 | 1.11 (1.04-1.18) | 0.002 | 1 (0.94-1.06) | 0.967 | 1.05 (0.99-1.12) | 0.088 |
|  | >90-105 | 1 (Ref) | NA | 1 (Ref) | NA | 1 (Ref) | NA |
|  | >75-90 | 0.95 (0.91-0.99) | 0.025 | 1.07 (1.03-1.12) | 0.002 | 1.02 (0.98-1.07) | 0.353 |
|  | >60-75 | 1.03 (0.97-1.09) | 0.335 | 1.19 (1.12-1.25) | <0.001 | 1.12 (1.06-1.19) | <0.001 |
|  | >45-60 | 1.05 (0.94-1.18) | 0.402 | 1.47 (1.35-1.6) | <0.001 | 1.36 (1.22-1.51) | <0.001 |
|  | >30-45 | 1.22 (0.94-1.59) | 0.133 | 1.66 (1.4-1.96) | <0.001 | 1.37 (1.08-1.73) | 0.009 |
|  | <=30 | 1.46 (0.84-2.52) | 0.176 | 1.7 (1.17-2.47) | 0.005 | 1.72 (1.11-2.68) | 0.016 |

eGFR: estimated glomerular filtration rate. Model 1 was adjusted for age, history of or medication for hypertension, systolic or diastolic blood pressure, haemoglobin and haematocrit. Model 2 was adjusted for variables included in Model 1, plus prescription of an anticoagulant (warfarin or low-molecular weight heparin) or an antiplatelet agent (aspirin, clopidogrel, prasugrel or dipyridamole) at baseline. Model 3 was adjusted for variables included in Model 2, plus ethnicity, body mass index and hip to waist ratio.

## Table S9

Cause-specific Cox proportional hazards model outputs in male participants for all-cause mortality

|  |  | **eGFRcr** |  | **eGFRcys** |  | **eGFRcrcys** |  |
| --- | --- | --- | --- | --- | --- | --- | --- |
| **Model** | **eGFR category**  **(mL/min/1.73m^2^)** | **HR (95% CI)** | **P value** | **HR (95% CI)** | **P value** | **HR (95% CI)** | **P value** |
| Model 1 | >105 | 1.71 (1.6-1.82) | <0.001 | 0.93 (0.87-1) | 0.044 | 1.12 (1.04-1.2) | 0.001 |
|  | >90-105 | 1 (Ref) | NA | 1 (Ref) | NA | 1 (Ref) | NA |
|  | >75-90 | 0.92 (0.88-0.95) | <0.001 | 1.14 (1.09-1.19) | <0.001 | 1.14 (1.09-1.18) | <0.001 |
|  | >60-75 | 1.02 (0.97-1.07) | 0.416 | 1.49 (1.42-1.56) | <0.001 | 1.41 (1.34-1.48) | <0.001 |
|  | >45-60 | 1.45 (1.35-1.57) | <0.001 | 2.2 (2.07-2.34) | <0.001 | 2.05 (1.9-2.2) | <0.001 |
|  | >30-45 | 2.24 (1.96-2.55) | <0.001 | 3.48 (3.18-3.82) | <0.001 | 3.05 (2.72-3.41) | <0.001 |
|  | <=30 | 3.54 (2.81-4.46) | <0.001 | 5.81 (4.97-6.8) | <0.001 | 5.04 (4.18-6.09) | <0.001 |
| Model 2 | >105 | 1.7 (1.59-1.82) | <0.001 | 0.93 (0.87-1) | 0.041 | 1.12 (1.04-1.2) | 0.002 |
|  | >90-105 | 1 (Ref) | NA | 1 (Ref) | NA | 1 (Ref) | NA |
|  | >75-90 | 0.92 (0.88-0.95) | <0.001 | 1.14 (1.09-1.19) | <0.001 | 1.14 (1.09-1.18) | <0.001 |
|  | >60-75 | 1.02 (0.97-1.07) | 0.403 | 1.49 (1.42-1.56) | <0.001 | 1.41 (1.34-1.47) | <0.001 |
|  | >45-60 | 1.44 (1.34-1.56) | <0.001 | 2.19 (2.06-2.33) | <0.001 | 2.03 (1.89-2.18) | <0.001 |
|  | >30-45 | 2.25 (1.98-2.57) | <0.001 | 3.47 (3.17-3.81) | <0.001 | 3.06 (2.73-3.42) | <0.001 |
|  | <=30 | 3.53 (2.81-4.45) | <0.001 | 5.89 (5.03-6.89) | <0.001 | 5.07 (4.2-6.12) | <0.001 |
| Model 3 | >105 | 1.71 (1.6-1.83) | <0.001 | 0.94 (0.88-1.01) | 0.101 | 1.13 (1.06-1.21) | 0.001 |
|  | >90-105 | 1 (Ref) | NA | 1 (Ref) | NA | 1 (Ref) | NA |
|  | >75-90 | 0.92 (0.89-0.96) | <0.001 | 1.12 (1.08-1.17) | <0.001 | 1.12 (1.08-1.17) | <0.001 |
|  | >60-75 | 1.02 (0.97-1.07) | 0.484 | 1.45 (1.38-1.52) | <0.001 | 1.37 (1.31-1.44) | <0.001 |
|  | >45-60 | 1.43 (1.32-1.54) | <0.001 | 2.1 (1.98-2.24) | <0.001 | 1.95 (1.82-2.1) | <0.001 |
|  | >30-45 | 2.17 (1.9-2.48) | <0.001 | 3.34 (3.05-3.67) | <0.001 | 2.94 (2.62-3.29) | <0.001 |
|  | <=30 | 3.5 (2.78-4.41) | <0.001 | 5.78 (4.94-6.76) | <0.001 | 4.91 (4.07-5.93) | <0.001 |

eGFR: estimated glomerular filtration rate. Model 1: for accepted age, smoking, systolic and diastolic blood pressure, medications for blood pressure or cholesterol, baseline total, LDL and HDL cholesterol, pre-existing heart failure, atrial fibrillation/flutter or other atherosclerotic cardiovascular disease (including myocardial infarction and peripheral vascular disease). Model 2 was adjusted for variables included in Model 1, plus prescription of an anticoagulant (warfarin or low-molecular weight heparin) or an antiplatelet agent (aspirin, clopidogrel, prasugrel or dipyridamole) at baseline. Model 3 was adjusted for variables included in Model 2, plus ethnicity, body mass index and hip to waist ratio.

## Table S10

Cause-specific Cox proportional hazards model outputs in female participants for all-cause mortality

|  |  | **eGFRcr** |  | **eGFRcys** |  | **eGFRcrcys** |  |
| --- | --- | --- | --- | --- | --- | --- | --- |
| **Model** | **eGFR category**  **(mL/min/1.73m^2^)** | **HR (95% CI)** | **P value** | **HR (95% CI)** | **P value** | **HR (95% CI)** | **P value** |
| Model 1 | >105 | 1.31 (1.2-1.42) | <0.001 | 0.84 (0.77-0.93) | 0.001 | 0.98 (0.91-1.07) | 0.708 |
|  | >90-105 | 1 (Ref) | NA | 1 (Ref) | NA | 1 (Ref) | NA |
|  | >75-90 | 0.94 (0.9-0.98) | 0.004 | 1.17 (1.11-1.23) | <0.001 | 1.13 (1.07-1.18) | <0.001 |
|  | >60-75 | 1.06 (1.01-1.12) | 0.028 | 1.43 (1.35-1.51) | <0.001 | 1.36 (1.29-1.44) | <0.001 |
|  | >45-60 | 1.35 (1.23-1.48) | <0.001 | 2.16 (2.01-2.32) | <0.001 | 1.93 (1.77-2.1) | <0.001 |
|  | >30-45 | 2.52 (2.13-2.99) | <0.001 | 3.42 (3.04-3.84) | <0.001 | 3.29 (2.84-3.82) | <0.001 |
|  | <=30 | 5.24 (3.92-7.02) | <0.001 | 7.75 (6.37-9.43) | <0.001 | 6.96 (5.49-8.83) | <0.001 |
| Model 2 | >105 | 1.31 (1.2-1.42) | <0.001 | 0.84 (0.77-0.93) | <0.001 | 0.98 (0.91-1.07) | 0.692 |
|  | >90-105 | 1 (Ref) | NA | 1 (Ref) | NA | 1 (Ref) | NA |
|  | >75-90 | 0.94 (0.9-0.98) | 0.004 | 1.17 (1.11-1.23) | <0.001 | 1.13 (1.07-1.18) | <0.001 |
|  | >60-75 | 1.06 (1-1.12) | 0.039 | 1.43 (1.35-1.51) | <0.001 | 1.36 (1.28-1.43) | <0.001 |
|  | >45-60 | 1.34 (1.22-1.47) | <0.001 | 2.15 (2-2.31) | <0.001 | 1.91 (1.75-2.08) | <0.001 |
|  | >30-45 | 2.47 (2.09-2.93) | <0.001 | 3.37 (3-3.78) | <0.001 | 3.24 (2.79-3.76) | <0.001 |
|  | <=30 | 5.23 (3.91-6.99) | <0.001 | 7.71 (6.34-9.39) | <0.001 | 6.95 (5.48-8.82) | <0.001 |
| Model 3 | >105 | 1.29 (1.18-1.4) | <0.001 | 0.85 (0.77-0.93) | 0.001 | 0.99 (0.91-1.07) | 0.782 |
|  | >90-105 | 1 (Ref) | NA | 1 (Ref) | NA | 1 (Ref) | NA |
|  | >75-90 | 0.94 (0.9-0.98) | 0.003 | 1.16 (1.1-1.22) | <0.001 | 1.11 (1.06-1.17) | <0.001 |
|  | >60-75 | 1.05 (0.99-1.11) | 0.098 | 1.41 (1.33-1.49) | <0.001 | 1.33 (1.26-1.41) | <0.001 |
|  | >45-60 | 1.32 (1.2-1.45) | <0.001 | 2.12 (1.97-2.28) | <0.001 | 1.86 (1.7-2.03) | <0.001 |
|  | >30-45 | 2.42 (2.05-2.87) | <0.001 | 3.34 (2.97-3.75) | <0.001 | 3.17 (2.73-3.69) | <0.001 |
|  | <=30 | 5.04 (3.75-6.76) | <0.001 | 7.54 (6.17-9.21) | <0.001 | 6.74 (5.29-8.58) | <0.001 |

eGFR: estimated glomerular filtration rate. Model 1: for accepted age, smoking, systolic and diastolic blood pressure, medications for blood pressure or cholesterol, baseline total, LDL and HDL cholesterol, pre-existing heart failure, atrial fibrillation/flutter or other atherosclerotic cardiovascular disease (including myocardial infarction and peripheral vascular disease). Model 2 was adjusted for variables included in Model 1, plus prescription of an anticoagulant (warfarin or low-molecular weight heparin) or an antiplatelet agent (aspirin, clopidogrel, prasugrel or dipyridamole) at baseline. Model 3 was adjusted for variables included in Model 2, plus ethnicity, body mass index and hip to waist ratio.

## Table S11

Baseline characteristics in the subgroup with atrial fibrillation/flutter at baseline

|  | **Female** | **Male** | **p** |
| --- | --- | --- | --- |
| N | 1,943 | 4,588 |  |
| Age (median [IQR]) | 64.00 [60.00, 67.00] | 63.00 [59.00, 67.00] | 0.08 |
| Ethnicity: n (%) | |  | 0.051 |
| White | 1886 (97.1) | 4477 (97.6) |  |
| Mixed | 4 (0.2) | 11 (0.2) |  |
| Black | 21 (1.1) | 22 (0.5) |  |
| South Asian | 11 (0.6) | 40 (0.9) |  |
| Chinese | 2 (0.1) | 6 (0.1) |  |
| Other | 10 (0.5) | 11 (0.2) |  |
| Unknown | 9 (0.5) | 21 (0.5) |  |
| Smoking: n (%) | |  | <0.001 |
| Never | 1051 (54.1) | 1892 (41.2) |  |
| Previous | 784 (40.3) | 2315 (50.5) |  |
| Current | 91 (4.7) | 357 (7.8) |  |
| Unknown | 17 (0.9) | 24 (0.5) |  |
| Systolic BP (mean (SD)) | 140.05 (21.32) | 140.01 (20.17) | 0.933 |
| Diastolic BP (mean (SD)) | 80.30 (11.94) | 83.01 (11.85) | <0.001 |
| Body mass index (mean (SD)) | 28.92 (6.12) | 29.06 (4.91) | 0.36 |
| Hip:waist ratio (mean (SD)) | 1.21 (0.10) | 1.06 (0.08) | <0.001 |
| eGFRcr (mL/min/1.73m^2^)  (mean (SD)) | 85.36 [72.34, 94.08] | 86.59 [74.42, 94.03] | 0.137 |
| eGFRcys (median [IQR]) | 77.46 [65.18, 89.48] | 77.99 [66.47, 90.37] | 0.006 |
| eGFRcrcys (mL/min/1.73m^2^)  (mean (SD)) | 81.19 [69.83, 91.30] | 82.17 [72.05, 91.90] | 0.02 |
| Urine albumin:creatinine ratio (median [IQR]) | 0.00 [0.00, 1.18] | 0.00 [0.00, 1.30] | <0.001 |
| Total cholesterol (mean (SD)) | 5.55 (1.19) | 4.89 (1.12) | <0.001 |
| LDL cholesterol (mean (SD)) | 3.40 (0.91) | 3.04 (0.84) | <0.001 |
| HDL cholesterol (mean (SD)) | 1.51 (0.38) | 1.22 (0.31) | <0.001 |
| C-reactive protein mg/L (mean (SD)) | 3.66 (5.42) | 3.21 (5.57) | 0.003 |
| Haemoglobin (g/dL) (mean (SD)) | 13.56 (1.04) | 14.90 (1.19) | <0.001 |
| Haematocrit (%) (mean (SD)) | 39.51 (3.06) | 43.17 (3.46) | <0.001 |
| Diabetes: n (%) | 175 (9.0) | 622 (13.6) | <0.001 |
| Hypertension: n (%) | 674 (34.7) | 1425 (31.1) | 0.004 |
| Cardiovascular disease: n (%) | 296 (15.2) | 1279 (27.9) | <0.001 |
| Heart failure: n (%) | 136 (7.0) | 637 (13.9) | <0.001 |
| Medications for blood pressure: n (%) | 379 (19.5) | 784 (17.1) | 0.021 |
| Medications for cholesterol: n (%) | 737 (37.9) | 2407 (52.5) | <0.001 |
| Anticoagulant: n (%) | 608 (31.3) | 1719 (37.5) | <0.001 |
| Antiplatelet: n (%) | 819 (42.2) | 2096 (45.7) | 0.009 |
| CHADS-VASC (median [IQR]) | 2.00 [2.00, 3.00] | 1.00 [1.00, 2.00] | <0.001 |
| ORBIT (median [IQR]) | 2.00 [0.00, 3.00] | 1.00 [0.00, 1.00] | <0.001 |

## Table S12

Proportion of people with pre-existing atrial fibrillation/flutter prescribed preventative medications by sex and CHADS-VASC score.

|  | **CHADS-VASC score** | | | |
| --- | --- | --- | --- | --- |
|  | **0** | **1** | **2** | **>3** |
| **Male** | | | | |
| N | 841 | 1,831 | 1,490 | 426 |
| Age (years): median [IQR] | 59.0 [54.0, 62.0] | 62.0 [58.0, 65.0] | 66.0 [64.2, 68.0] | 67.0 [65.0, 68.0] |
| eGFRcr (mL/min/1.73m^2^): median [IQR] | 96.9 [85.5, 101.8] | 93.7 [81.0, 99.8] | 87.9 [75.4, 95.7] | 83.5 [68.9, 95.1] |
| Hypertension: n(%) | 0 (0.0) | 615 (33.6) | 650 (43.6) | 160 (37.6) |
| Diabetes: n (%) | 0 (0.0) | 69 (3.8) | 278 (18.7) | 275 (64.6) |
| Heart failure: n (%) | 0 (0.0) | 131 (7.2) | 290 (19.5) | 216 (50.7) |
| Statin: n (%) | 291 (34.6) | 864 (47.2) | 919 (61.7) | 333 (78.2) |
| Antiplatelet: n (%) | 421 (50.1) | 828 (45.2) | 672 (45.1) | 175 (41.1) |
| Anticoagulation: n (%) | 202 (24.0) | 654 (35.7) | 633 (42.5) | 230 (54.0) |
| **Female** | | | | |
| N | 0 | 414 | 768 | 761 |
| Age (years): median [IQR] | - | 60.0 [54.0, 62.0] | 62.0 [59.0, 65.0] | 67.0 [65.0, 68.0] |
| eGFRcr (mL/min/1.73m^2^): median [IQR] | - | 94.6 [83.9, 102.0] | 91.5 [78.9, 99.6] | 84.6 [71.8, 96.3] |
| Hypertension: n(%) | - | 0 (0.0) | 289 (37.6) | 385 (50.6) |
| Diabetes: n (%) | - | 0 (0.0) | 21 (2.7) | 154 (20.2) |
| Heart failure: n (%) | - | 0 (0.0) | 33 (4.3) | 103 (13.5) |
| Statin: n (%) | - | 101 (24.4) | 243 (31.6) | 393 (51.6) |
| Antiplatelet: n (%) | - | 175 (42.3) | 314 (40.9) | 330 (43.4) |
| Anticoagulation: n (%) | - | 91 (22.0) | 221 (28.8) | 296 (38.9) |

## Figure S1

Density plot showing the differences in eGFRcr, eGFRcys and eGFRcrcys by sex in the UK Biobank population

## Figure S2

In a sub-population of participants with atrial fibrillation/flutter at baseline, forest plots displaying cause-specific hazard ratio and 95% confidence intervals (95% CI) for ischaemic stroke (left), haemorrhagic stroke (middle) or any major bleeding (right). Results taken from Cox proportional hazards models censored for death not caused by the outcome of interest. Ischaemic stroke models were adjusted for CHADSVASC score. Haemorrhagic stroke and major bleeding models were adjusted for ORBIT score. All models were stratified by sex.
